# Thermal stability and diffusion characteristics of ultrathin amorphous carbon films grown on crystalline and nitrogenated silicon substrates by filtered cathodic vacuum arc deposition

**DOI:** 10.1038/s41598-021-91903-y

**Published:** 2021-06-23

**Authors:** Shengxi Wang, Anurag Roy, Kyriakos Komvopoulos

**Affiliations:** Department of Mechanical Engineering, University of California, Berkeley, CA 94720 USA

**Keywords:** Materials science, Nanoscience and technology

## Abstract

Amorphous carbon (*a*-C) films are widely used as protective overcoats in many technology sectors, principally due to their excellent thermophysical properties and chemical inertness. The growth and thermal stability of sub-5-nm-thick *a*-C films synthesized by filtered cathodic vacuum arc on pure (crystalline) and nitrogenated (amorphous) silicon substrate surfaces were investigated in this study. Samples of *a*-C/Si and *a*-C/SiN_x_/Si stacks were thermally annealed for various durations and subsequently characterized by high-resolution transmission electron microscopy (TEM) and electron energy loss spectroscopy (EELS). The TEM images confirmed the continuity and uniformity of the *a*-C films and the 5-nm-thick SiN_x_ underlayer formed by silicon nitrogenation using radio-frequency sputtering. The EELS analysis of cross-sectional samples revealed the thermal stability of the *a*-C films and the efficacy of the SiN_x_ underlayer to prevent carbon migration into the silicon substrate, even after prolonged heating. The obtained results provide insight into the important attributes of an underlayer in heated multilayered media for preventing elemental intermixing with the substrate, while preserving the structural stability of the *a*-C film at the stack surface. An important contribution of this investigation is the establishment of an experimental framework for accurately assessing the thermal stability and elemental diffusion in layered microstructures exposed to elevated temperatures.

## Introduction

Amorphous carbon (*a*-C) films are used as protective overcoats in various applications, predominantly because of their unique physical properties, such as chemical inertness, thermal stability, optoelectrical characteristics, tribomechanical properties, biocompatibility, and corrosion resistance^1–4^. With the exception of optoelectrical characteristics, all of the foregoing properties of *a*-C films are linked to a high fraction of tetrahedral (*sp*^3^) carbon atom hybridization. Among various techniques of *a*-C film deposition^5–8^, the most commonly used methods are plasma-enhanced chemical vapor deposition, ion beam deposition, radio-frequency (RF) and magnetron sputtering, pulsed laser deposition, and filtered cathodic vacuum arc (FCVA). Particularly, the FCVA technique has been proven to be one of the most effective methods for synthesizing ultrathin *a*-C films with high *sp*^3^ contents. The growth of *sp*^3^-rich *a*-C films using the FCVA method is attributed to several intrinsic process features, such as low-temperature film growth, swift plasma manipulation, effective macroparticle filtering, pulsed substrate biasing, and stable plasma arcing^9^. These advantages have made FCVA the prime process of *a*-C film deposition in several thin-film technology sectors, including magnetic storage devices, laser photonics, microelectromechanical systems, and biomedical implants^2, 4, 10–12^.

It is well established that *a*-C films grown by deposition methods wherein the film precursors are energetic particles, such as C^+^ ions in carbon film deposition by the FCVA process, exhibit a layered structure architecture consisting of three main layers, i.e., intermixing, bulk, and surface layers^13^, with the intermediate *sp*^3^-rich bulk layer being mainly responsible for the high hardness, excellent tribomechanical properties, and good thermal stability demonstrated by FCVA-deposited *a*-C films^14, 15^. The substrate bias voltage controls the energy of the C^+^ ions impinging onto the substrate and film surfaces. The implantation of energetic C^+^ ions into the film/substrate system, a process known as subplantation^16^, leads to the formation of an intermixing layer consisting of film and substrate atoms, the densification of the growing film, and the development of an *sp*^3^-rich bulk layer in the core of the *a*-C film. The thickness of the intermixing layer depends on the penetration range of the incident C^+^ ions, which is controlled by the substrate bias voltage, whereas the thickness of the predominantly *sp*^3^-hybridized bulk layer varies with the *a*-C film thickness. The formation of a relatively low density, principally trigonally hybridized (*sp*^2^) surface layer is due to the limited ion bombardment of the carbon material deposited onto the outermost region of the film during the final stage of film growth.

In concurrence with the synthesis of ultrathin *a*-C films, specialized characterization methods were also developed in response to high demands for imaging techniques that can elucidate the composition and structure of films with thicknesses in the low nanometer range. The ion incidence angle, substrate bias voltage, and duty cycle of pulse biasing strongly affect the thickness, composition, and structure of *a*-C films grown by the FCVA deposition method^17–20^. Deciphering the local nanostructure and ascertaining the chemical composition at the nanoscale have enabled more accurate estimations of the *sp*^2^ and *sp*^3^ fractions and the thickness of individual layers comprising the layered structure of ultrathin *a*-C films^13, 18–22^. The overall *sp*^3^ content of an *a*-C film is of paramount importance because it directly affects the thermal, structural, and tribomechanical properties of the film and the magnitude of the intrinsic compressive stress^14, 23^, which is conducive to *sp*^3^ hybridization.

In conjunction with contemporary trends for device miniaturization, the thickness of protective films has been constantly decreasing, raising concerns about maintaining the physical properties that are intrinsic of the film’s protective capability, such as uniformity, density, wear and corrosion resistance, and thermal stability. For example, the thickness of *a*-C films used as protective overcoats in current data storage hard-disk drives is in the range of 2–4 nm^20, 24^. A comparison of the results reported in previous studies^13, 19, 20^ reveals a monotonic decrease of the thickness of the *sp*^3^-rich bulk layer with decreasing *a*-C film thickness. Accordingly, excessive thinning may seriously compromise the protective competency of the *a*-C film. This problem is further exacerbated in elevated-temperature applications, such as laser photonics and heat-assisted magnetic recording (HAMR). However, despite significant insight into the structure of ultrathin *a*-C films exposed to ambient conditions derived from previous studies^18–20, 25, 26^, relatively less is known about the structural stability of ultrathin *a*-C films at elevated temperatures. Since several technologies rely on effective device operation at elevated temperatures, maintaining the mechanical integrity of *a*-C films in hot environments is critical. For example, *a*-C films used to protect vital head components of HAMR hard-disk drives, such as the near-field transducer and the read/write pole, must demonstrate structural stability at temperatures in the range of 100–300 ^o^C^27^. Transient thermal spikes and prolonged heating may destabilize an ultrathin *a*-C film^28^. To examine this important issue, thermal annealing studies were performed to illuminate temperature-induced changes in the structure and composition of ultrathin *a*-C films^28–32^. Nevertheless, the carbon films examined in the foregoing studies were subjected to either short-time thermal annealing^28, 30, 31^ or laser-induced localized heating^29, 32^, were either hydrogenated^30^ or deposited on FePt magnetic media of hard disks^29, 32^, and were characterized by XRR, XPS, and Raman techniques^29–32^, which are not effective when the film thickness is less than 5 nm due to fundamental limitations. Specifically, XRR cannot accurately measure the density of < 20-nm-thick films; XPS uses the chemical shift between *sp*^2^ and *sp*^3^ components that are fitted to the C1s spectrum to obtain information about the material structure, thus yielding information about the overall composition up to a depth of  ~10 nm, which is much larger than the thickness of the films examined herein; and Raman is only sensitive to *sp*^2^ hybridization and is usually used to indirectly interpret the *sp*^2^/*sp*^3^ ratio by the first-order D-to-G band ratio, which lacks accuracy, especially when applied to ultrathin films. More importantly, none of the former characterization methods can yield through-thickness depth profiles of the hybridization state, which is critical when the spatial variation of hybridization is of interest, and cannot accurately determine the interfaces of the intermixing, bulk, and surfaces layers comprising the layered structure of ultrathin *a*-C films.

Furthermore, in applications involving prolonged exposure to an elevated temperature, such as a HAMR magnetic head, oxidation, delamination, *sp*^3^ → *sp*^2^ rehybridization (graphitization), and carbon diffusion into the substrate may compromise the reliability of the data storage device. Accordingly, an underlayer can be extremely beneficial not only for preventing carbon diffusion, especially if the intermixing layer of the *a*-C film cannot fulfil the role of a diffusion barrier, but also for acting as an adhesive and anti-corrosion layer that can effectively bond the *a*-C film to the substrate and prevent corrosion on the metallic substrate^26, 33–35^. However, none of the former studies investigated the thermal stability and, particularly, the diffusion characteristics of *a*-C films in the presence of an adhesion underlayer under conditions of prolonged heating. Specifically, rapid heating (1 s) was used to evaluate the thermal stability of *a*-C films intended for HAMR hard-disk overcoats^28^ and short-duration (2 or 10 min) thermal annealing experiments were carried out to assess the structural stability of diamond-like carbon films^31^, thus completely changing the dynamics of the diffusion process. Moreover, although there have been some studies dealing with the thermal stability of ultrathin *a*-C films^32^, no previous study has exclusively examined the thermal stability, and, particularly, the diffusion characteristics of *a*-C films in the presence of a SiN_x_ underlayer and under conditions of prolonged heating. In addition, aside from the short heating time used in earlier studies^28, 31, 32^, the focus was on changes in carbon films intended for the hard disks, not the magnetic head of HAMR drives, which is exposed to prolonged heating during the steady-state operation of the drive.

In view of the aforementioned challenges and gaps in scientific insight, the present study was undertaken with the main objective of exploring the efficacy of an ultrathin SiN_x_ underlayer to prevent carbon diffusion into the substrate, while preserving the thermal stability of ultrathin *a*-C films synthesized under optimal FCVA deposition conditions. Rapid thermal annealing (RTA) experiments were performed at an elevated temperature for extended durations to gauge two phenomena, i.e., the ability of the SiN_x_ underlayer to prevent carbon diffusion into the substrate and the thermal stability of the < 5-nm-thick *a*-C film. High-resolution transmission electron microscopy (TEM) and electron energy loss spectroscopy (EELS) were used to evaluate the continuity, thickness, and hybridization state of the layers comprising the multilayered *a*-C/Si and *a*-C/SiN_x_/Si stacks and the through-thickness structure of the intermixing, bulk, and surface layers of the *a*-C film before and after the RTA treatment. Results from the TEM and EELS studies of the *a*-C/Si and *a*-C/SiN_x_/Si stacks are contrasted below to assess the aforementioned competencies of the SiN_x_ underlayer and to illuminate its effect on the thermal stability of the *a*-C film. The novelty of the present study is the elucidation of the spatial distribution of carbon and *sp*^3^ hybridization through the thickness (depth profiles) of the layered *a*-C film structure before and after thermal annealing and the diffusion range of carbon into the Si substrate and SiN_x_ underlayer of the *a*-C/Si and *a*-C/SiN_x_/Si stacks, respectively, revealing the efficacy of the SiN_x_ underlayer to prevent carbon diffusion into the substrate material.

## Results and discussion

**Figure 1:**
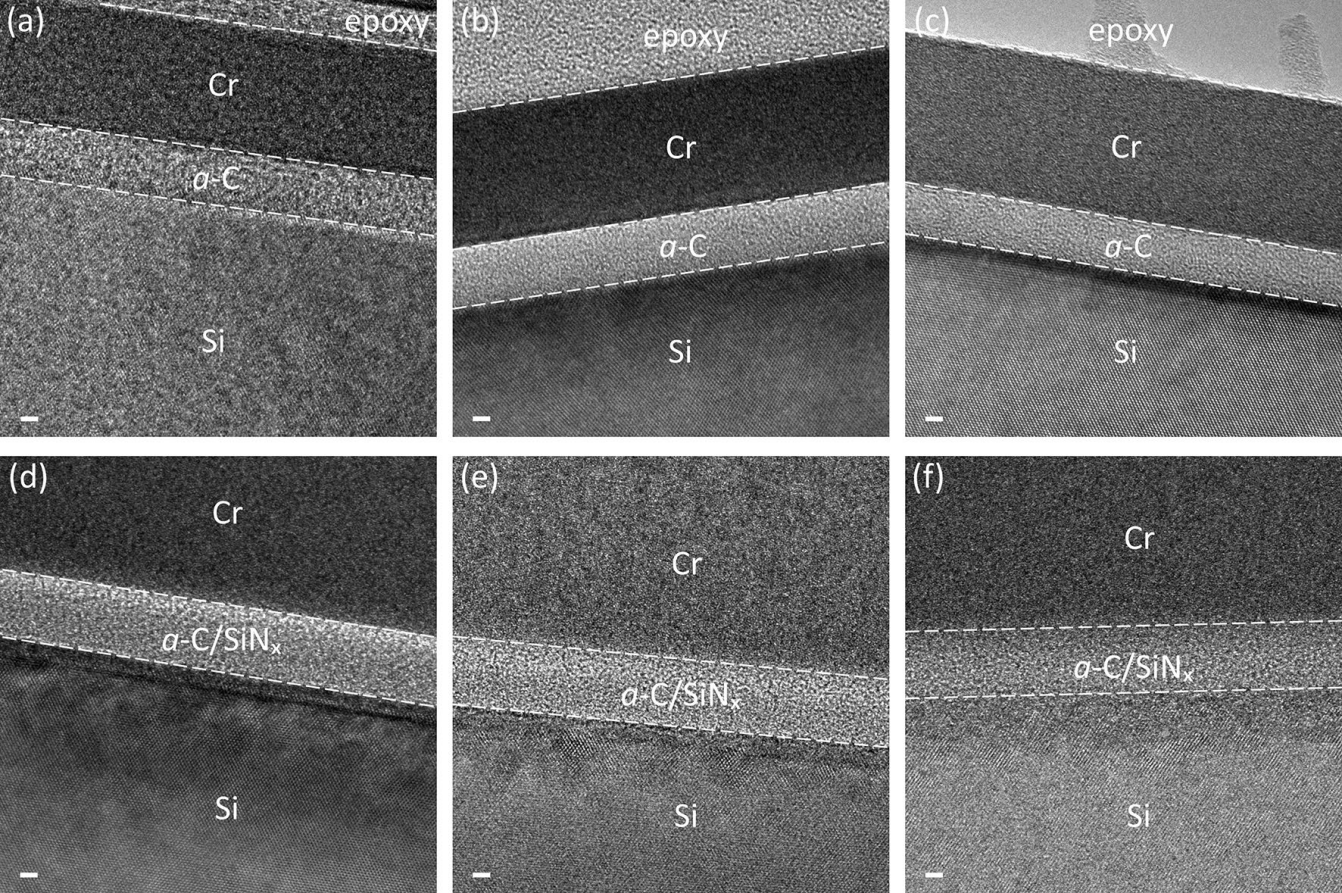
Cross-sectional TEM images of (**a**–**c**) *a*-C/Si and (**d**–**f**) *a*-C/SiN_x_/Si stacks obtained before (**a**,**d**) and after RTA at 250 °C in Ar atmosphere for (**b**,**e**) 30 min and (**c**,**f**) 90 min. All scale bars are equal to 2 nm.

Figure 1 shows representative cross-sectional TEM images of *a*-C/Si and *a*-C/SiN_x_/Si stacks obtained before and after RTA for 30 and 90 min. While the crystalline Si substrate exhibits long-range atomic order, the *a*-C film and the SiN_x_ underlayer are both amorphous; consequently, distinguishing them based on contrast differences is difficult, especially because they are adjacent layers in the stack. Thus, a SiN_x_/Si sample was used to determine the nominal thickness of the SiN_x_ underlayer based on contrast differences in TEM imaging. Cross-sectional TEM images revealed that the nominal thickness of the *a*-C film in the *a*-C/Si and *a*-C/SiN_x_/Si stacks was equal to 4.8 ± 0.8 nm and 2.7 ± 0.3 nm, respectively, whereas that of the SiN_x_ underlayer in the *a*-C/SiN_x_/Si stack was equal to 5.0 ± 0.2 nm. The different thicknesses of the *a*-C film in the two stacks is partly attributed to differences in the sticking coefficient of C on Si and SiN_x_ as well as the subplantation effect. Because the layer thickness measurements obtained from the TEM images were based on qualitative visual contrast differences, they represent nominal thickness estimates. Consequently, accurate thickness measurements were obtained with a more precise method, which uses characteristic elements (i.e., Si, N, and C) in the EELS spectra that are chemical fingerprints of Si, SiN_x_, and *a*-C. Therefore, the main observation from the TEM images is that the synthesized *a*-C films are continuous, uniform, and demonstrate desired surface morphology attributes, such as high surface smoothness. In addition, the films are devoid of macroparticles, illustrative of the efficiency of the present FCVA setup to effectively filter out any macroparticles expelled from the graphite cathode during arcing.

**Figure 2:**
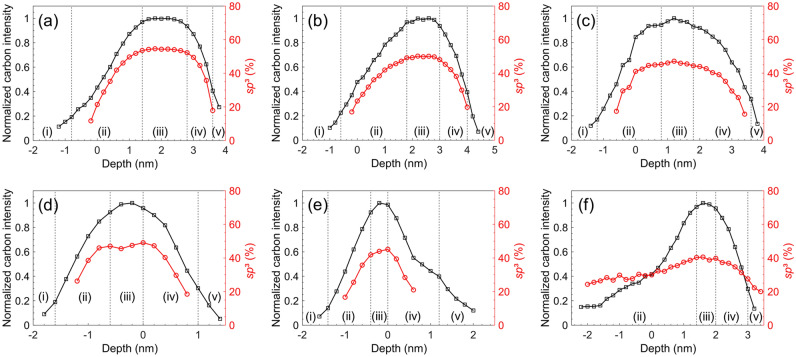
Depth profiles of normalized carbon intensity (black curves) and *sp*^3^ fraction (red curves) obtained from the C K-edge EELS spectrum of *a*-C films in (**a**–**c**) *a*-C/Si and (**d**–**f**) *a*-C/SiN_x_/Si stacks obtained before RTA (**a**,**d**) and after RTA at 250 °C in Ar atmosphere for (**b**,**e**) 30 min and (**c**,**f**) 90 min. The EELS profiles reveal a layered cross-sectional structure consisting of (i) substrate (Si or SiN_x_), (ii) intermixing layer, (iii) bulk layer, (iv) surface layer, and (v) capping layer (Cr).

**Figure 3:**
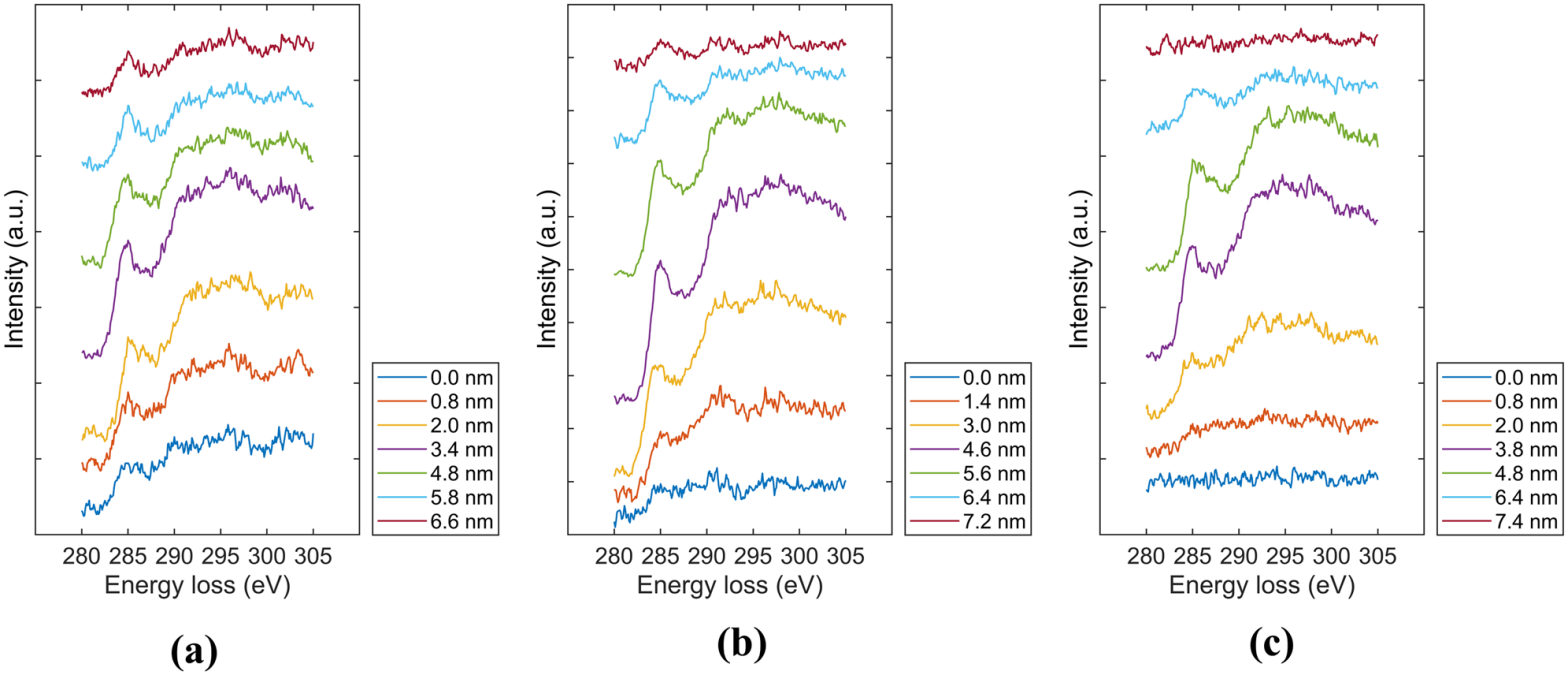
C K-edge EELS spectra of *a*-C/Si stacks: (**a**) untreated and (**b**,**c**) thermally annealed at 250 °C in Ar atmosphere for (**b**) 30 min and (**c**) 90 min. Representative spectra of the intermixing, bulk, and surface layers of the *a*-C film were obtained by advancing from the bottom spectra (blue color) of the Si substrate to the top spectra (dark red color) of the Cr capping layer in 0.2-nm increments.

**Figure 4:**
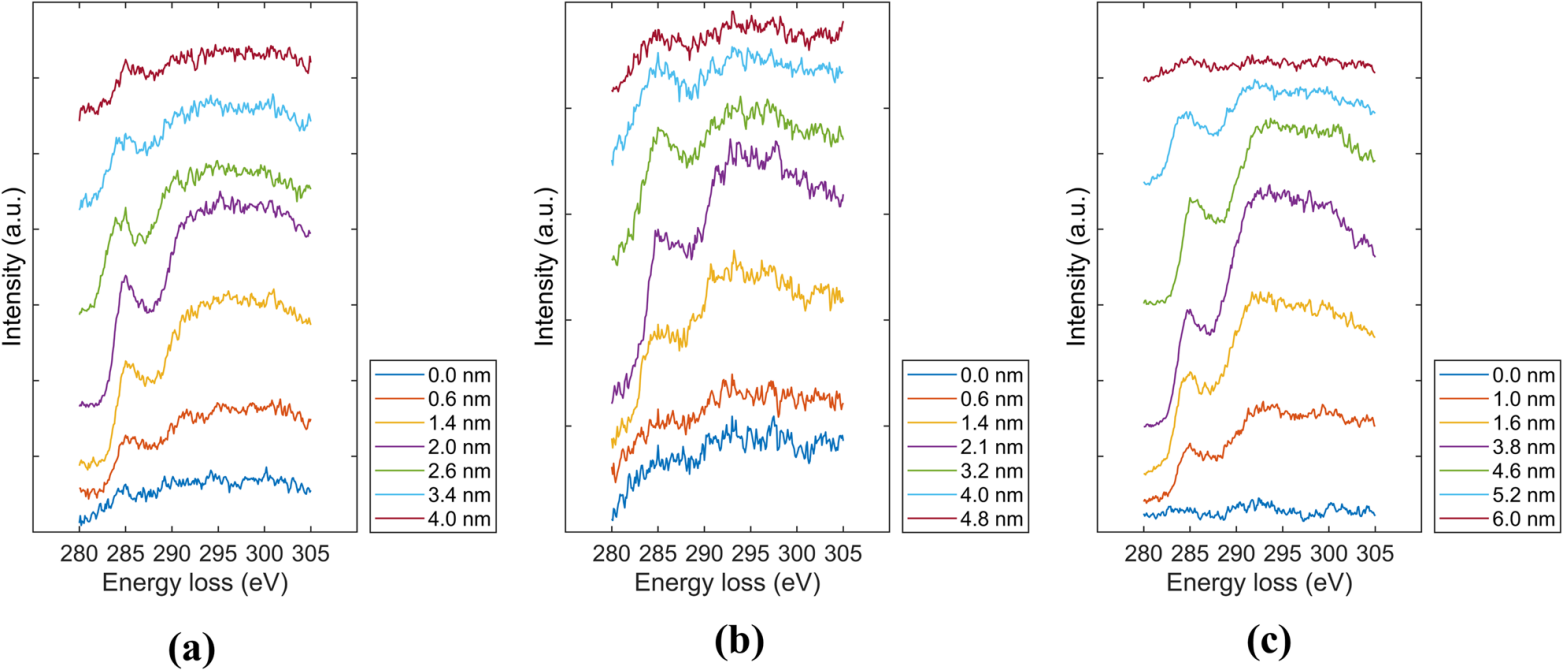
C K-edge EELS spectra of *a*-C/SiN_x_/Si stacks: (**a**) untreated and (**b**,**c**) thermally annealed at 250 °C in Ar atmosphere for (**b**) 30 min and (**c**) 90 min. Representative spectra of the intermixing, bulk, and surface layers of the *a*-C film were obtained by advancing from the bottom spectra (blue color) of the Si substrate to the top spectra (dark red color) of the Cr capping layer in 0.2-nm increments.

Figure 2 shows the normalized carbon intensity and the *sp*^3^ fraction estimated from C K-edge EELS spectra of the *a*-C/Si and *a*-C/SiN_x_/Si stacks before and after RTA for 30 and 90 min. The first detectable count of the C peak was assigned a depth value of –2 nm, which was used as the reference depth for the rest of the data of the carbon intensity and *sp*^3^ depth profiles. The carbon atom hybridization fractions are of paramount importance because the *sp*^2^ correlates with the optical and electrical properties, whereas the *sp*^3^ controls the hardness, tribological properties, and thermal stability of the *a*-C film. In addition, the EELS spectra provide an effective means of determining the layer interfaces in the stacks by a previously developed method^24^, accordingly enabling the accurate estimation of the thicknesses of the individual layers comprising the *a*-C film and the total thickness of the *a*-C film and the SiN_x_ underlayer. Specifically, the interface delineating the intermixing layer was assigned a normalized carbon intensity of < 0.15 in tandem with a sharply increasing *sp*^3^ fraction, the interface of the intermixing layer with the bulk layer was determined by a normalized carbon intensity of 0.9–1.0 and afterward approximately constant *sp*^3^ fraction, whereas the interface of the bulk layer with the surface layer was defined by a normalized carbon intensity of 0.85–0.95, followed by a sharp decrease of the *sp*^3^ fraction. Finally, the interface between the surface layer and the capping layer was identified as the location having a normalized carbon intensity of 0.3–0.4 and very low and constant *sp*^3^ fraction, attributed to adventitious carbon seeping into the matrix of the capping layer. Representative C K-edge EELS spectra of the untreated (as-fabricated) and thermally annealed *a*-C/Si and *a*-C/SiN_x_/Si stacks are respectively shown in Figs. 3 and 4. The collection of these spectra was accomplished by the incremental advancement of the electron beam from the Si substrate or the SiN_x_ underlayer toward the Cr capping layer.

Synthesizing ultrathin *a*-C films by deposition methods that use energetic particles as film precursors, such as the FCVA process, is challenging because, as mentioned earlier, reducing the film thickness leads to significant thinning of the *sp*^3^-rich bulk layer, which controls almost all of the aforementioned desirable attributes of the film. This problem becomes even more acute when the film is exposed to an elevated temperature that may activate *sp*^3^ → *sp*^2^ rehybridization and destabilize the structure of the film. Hence, a challenging task is to decrease the *a*-C film thickness without compromising its physical properties and structural integrity. A comparison of the EELS profiles shown in Fig. 2 reveals only a slight decrease in *sp*^3^ content due to the RTA treatment. In particular, for the *a*-C/Si stack (Fig. 2a–c), the *sp*^3^ content of the bulk layer decreased by 4.5% and 7.5% after RTA treatment for 30 and 90 min, respectively, whereas for the *a*-C/SiN_x_/Si stack, the respective decrease of the *sp*^3^ content of the bulk layer was 3.9% and 8.5%. These findings reveal a marginal decrease in *sp*^3^ content of the bulk layer of thermally treated *a*-C films, providing evidence of the thermal stability of the *a*-C film deposited on Si and SiN_x_ under the selected FCVA process conditions. The decrease of the *sp*^3^ fraction is attributed to the prolonged thermal annealing, which provided the carbon atoms with the energy needed to diffuse through the layered film structure and into the SiN_x_ underlayer or the Si substrate. Carbon diffusion relaxed the compressive mechanical environment in the bulk layer produced by the subplantation process, an intrinsic characteristic of FCVA deposition, activating a transition from metastable *sp*^3^ hybridization to thermodynamically favorable *sp*^2^ hybridization, which reduced the *sp*^3^ fraction of the bulk layer. As mentioned earlier, a comparison of the results reported in previous studies^13, 19, 20^ shows a continuous decrease of the thickness of the *sp*^3^-rich bulk layer with decreasing *a*-C film thickness. Thus, while the decrease of the bulk layer thickness due to the RTA treatment is ascribed to carbon diffusion into the intermixing layer, the thinner bulk layer of the *a*-C film in the *a*-C/SiN_x_/Si stack is attributed to the growth of a thinner *a*-C film in this stack, for the reasons explained earlier.

**Figure 5:**
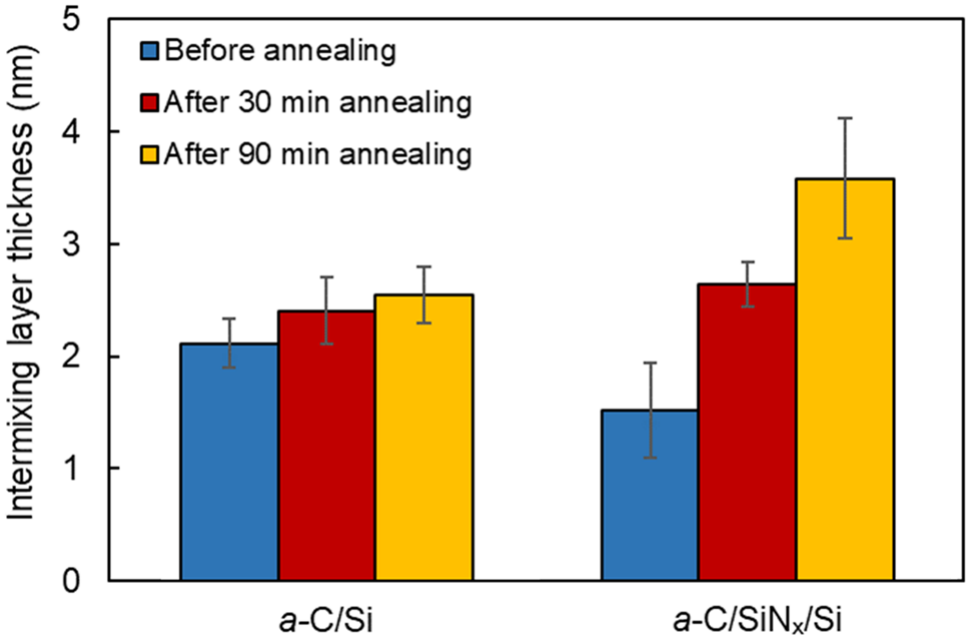
Intermixing layer thickness in *a*-C/Si and *a*-C/SiN_x_/Si stacks measured before and after RTA at 250 °C in Ar atmosphere for 30 and 90 min.

**Figure 6:**
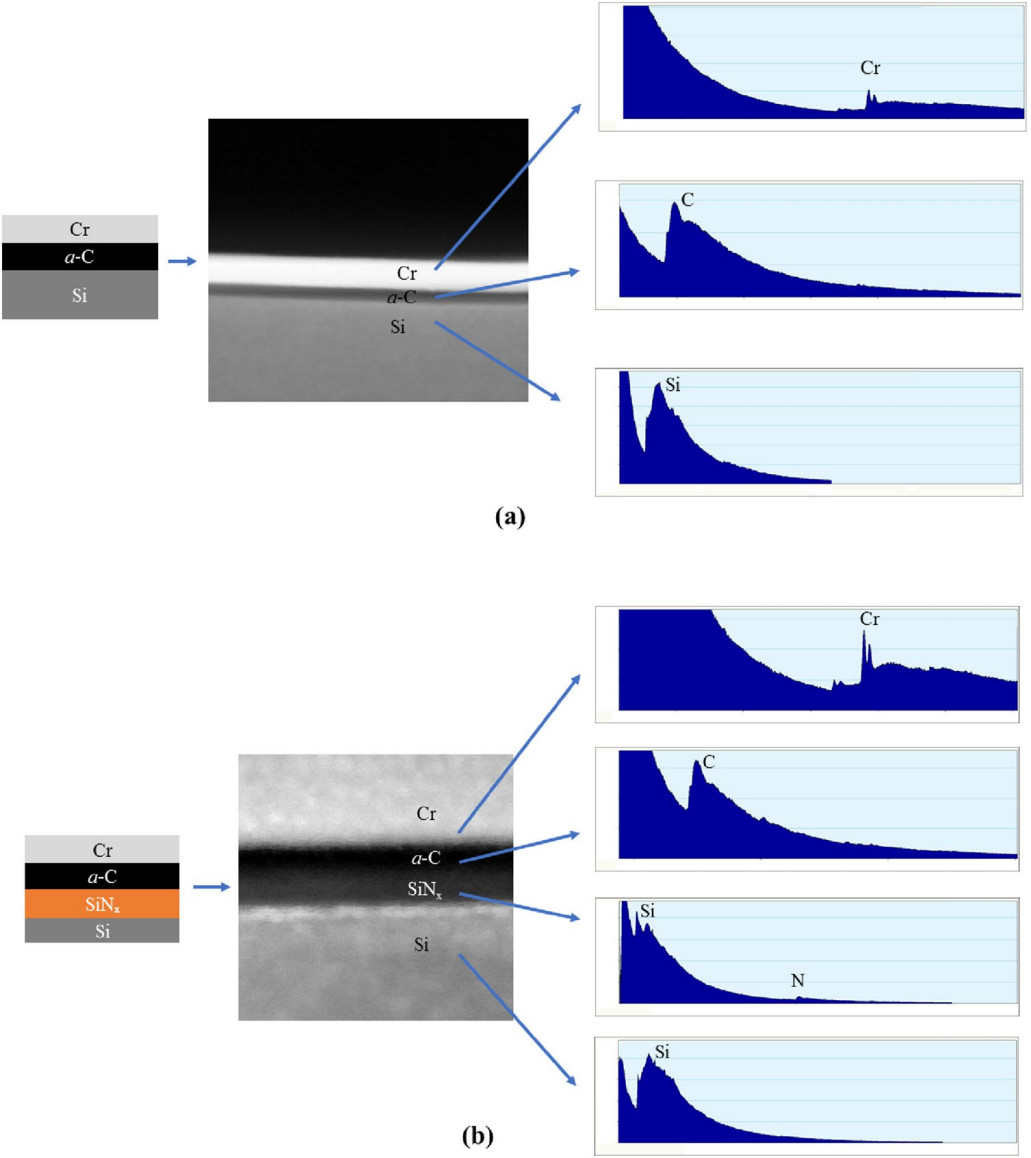
Schematic illustration of the (**a**) *a*-C/Si and (**b**) *a*-C/SiN_x_/Si stacks with the Cr capping layer on top of the overcoat and their corresponding STEM micrographs, accompanied by full survey EELS spectra of each layer present in the two layered stacks. (The vertical and horizontal axes in the EELS spectra represent counts and electron energy loss in eV, respectively).

Figure 5 shows the thickness of the intermixing layer, measured from EELS scans acquired along the cross section of the *a*-C/Si and *a*-C/SiN_x_/Si stacks before and after RTA for 30 and 90 min. EELS scanning was instigated at the crystalline Si substrate where only the L_2,3_-major edge peak was observed at 99 eV. By scanning across the stack cross section, the interface of the Si substrate with the SiN_x_ underlayer was revealed by the first appearance of the N K-major edge peak at 401 eV, that is, the SiN_x_ underlayer was identified by the firstly observed coexistence of the Si and N peaks in the EELS spectrum. Similarly, the interface of the SiN_x_ underlayer with the *a*-C film was determined by the first appearance of the C K-major edge peak at 285 eV, also designating the start of the intermixing layer up until the point that both the Si and N peaks disappeared from the EELS spectrum. Finally, the *a*-C film surface was determined by the last point where the C K-major edge peak was detected, accordingly enabling the precise calculation of the total *a*-C film thickness, i.e., the sum of the thicknesses of the intermixing, bulk, and surface layers. Beyond this point, only the Cr L_2,3_-major edge peak (attributed to the capping layer) could be detected in the range of 575–584 eV of the EELS spectrum. This procedure was used to compute the thicknesses of the *a*-C film and the SiN_x_ underlayer (Table 1) and the thickness of the intermixing layer in the *a*-C/Si and *a*-C/SiN_x_/Si stacks (Fig. 5). Full survey EELS spectra of characteristic elements (i.e., Si, C, and N) collected from cross-sectional TEM samples were used to determine the thickness of the intermixing layer in the *a*-C/Si and *a*-C/SiN_x_/Si stacks and the thickness of the SiN_x_ underlayer. The determination of the thickness of each layer with the foregoing method is preferred from that based on the visual inspection of the TEM images because it uses fingerprint elements of each material rather than contrast differences. Moreover, it was not possible to detect any changes in the thickness of the intermixing, bulk, and surface layers of the *a*-C film due to carbon migration instigated by the RTA treatment using the TEM images. Thus, all of the thickness measurements in this study were deduced from the EELS spectra, while the TEM images were only used to estimate the nominal thickness of the SiN_x_ underlayer and the *a*-C film (not including the intermixing layer, which was not discernible in the TEM images) and to obtain an overall assessment of the cross-sectional structure of the *a*-C/Si and *a*-C/SiN_x_/Si stacks. To illustrate the cross-sectional imaging and spectroscopy techniques utilized in this study, schematic graphics of the *a*-C/Si and *a*-C/SiN_x_/Si stacks are shown in Fig. 6 together with corresponding scanning TEM (STEM) micrographs and full survey EELS spectra of each layer present in the layered stacks. The trend of the total *a*-C film thickness (i.e., the sum of the intermixing, bulk, and surface layer thicknesses) to increase with the RTA duration, indicated by the data given in Table 1, is attributed to carbon diffusion into the Si substrate and the SiN_x_ underlayer of the *a*-C/Si and *a*-C/SiN_x_/Si stacks, respectively, which mainly increased the thickness of the intermixing layer (Fig. 5) and, in turn, the total thickness of the *a*-C film.

**Table 1:** Stack configurations and layer thickness estimated from EELS spectra acquired before and after RTA at 250 °C in Ar atmosphere for 30 and 90 min.

Substrate	Layer composition		Layer thickness (nm)					
			Before annealing		After annealing for 30 min		After annealing for 90 min	
	Layer 1	Layer 2	Layer 1	Layer 2	Layer 1	Layer 2	Layer 1	Layer 2
Si	–	*a*-C	–	5.6 ± 1.0	–	6.6 ± 0.4	–	7.0 ± 1.0
Si	SiN_x_	*a*-C	6.9 ± 0.4	3.4 ± 0.3	6.8 ± 0.2	4.0 ± 0.3	6.9 ± 0.4	5.4 ± 0.4

The dual role of the SiN_x_ underlayer is to enhance the adhesive strength of the *a*-C film to the substrate and to preserve the structure and composition of the substrate by inhibiting alloying due to carbon implantation during film deposition and/or diffusion at elevated temperatures. Figure 5 reveals that the SiN_x_ underlayer satisfied the latter objective. Indeed, a thinner intermixing layer fully confined within the SiN_x_ underlayer formed even after heating for 90 min. However, although the effect of the RTA treatment on the thickness of the intermixing layer in the *a*-C/Si stack was marginal, the thickness of the intermixing layer in the *a*-C/SiN_x_/Si stack increased with the RTA time. This can be explained by considering that the SiN_x_ underlayer was formed by nitrogenation of the Si substrate. The implanted N^+^ ions generated pathways for the carbon atoms to move through the SiN_x_ underlayer during the RTA treatment. Moreover, the valence electrons of most silicon atoms interacted with the implanted nitrogen atoms during the formation of the intermixing layer in the *a*-C/SiN_x_/Si stack, leaving significantly fewer sites for carbon atoms to bond during the growth of the *a*-C film, consequently enabling carbon implantation deeper into the SiN_x_ underlayer. Compared to the *a*-C/SiN_x_/Si stack, the carbon atoms in the intermixing layer of the *a*-C/Si stack had significantly more silicon atoms to bond to during direct and recoil implantation. Although this increased the intermixing layer thickness, it also inhibited carbon atom migration into the substrate during RTA, which explains the secondary RTA effect on the intermixing layer thickness in the *a*-C/Si stack. The thickness change of the *a*-C film, SiN_x_ underlayer, and intermixing layer reported herein are consistent with the findings of previous studies^22, 28^.

A principal objective of this study was to determine whether an ultrathin underlayer can inhibit carbon diffusion into the substrate during heating. It is noted that irrespective of the incorporation of an underlayer or not, carbon diffusion across the interfaces of the intermixing, bulk, and surface layers comprising the *a*-C film is inevitable at an elevated temperature. If a metallic substrate is used in the actual application (e.g., the FeNi alloy used to fabricate the read/write element of a HAMR head) instead of the silicon substrate used in this study, carbon diffusion may degrade some of the important substrate attributes, i.e., the magnetic properties of the FeNi alloy in this particular case. Thus, if the carbon atoms from the *a*-C overcoat were able to breach the SiN_x_ underlayer and diffuse into the substrate, it would imply the formation of metallic carbides, which would be detrimental to the magnetic properties of the head media. The EELS results of the present study provide a definitive proof that carbon diffusion from the *a*-C film into the substrate was prevented by arresting the diffusing carbon atoms inside the SiN_x_ underlayer.

In traditional physical and chemical vapor deposition processes, intermixing of the film with the substrate either does not occur or is very limited. As a consequence, there is a high probability of film delamination due to thermomechanical loadings, particularly for film/substrate systems characterized by a significant lattice mismatch and different elastic properties and thermal expansion coefficients. This major shortfall can be offset by a post-deposition thermal treatment that can promote elemental interdiffusion or, more effectively, by a deposition process like the FCVA that integrates the film with the substrate in the same process step through the formation of an intermixing layer. Because of the charged film precursors (e.g., C^+^ ions for *a*-C film growth), applying an optimal substrate bias voltage in FCVA deposition^14, 22^ leads to the formation of an intermixing layer with a thickness in the range of about 0.5–3 nm^19–21^ by the subplantation process^16^. This enhances the adhesive strength of the film to the substrate, while preserving the structure and composition of the substrate and suppressing the development of large strain gradients at the film/substrate interface. Therefore, the SiN_x_ underlayer not only augments the interfacial adhesive strength^25, 26, 33, 36^ but, as shown in this study, also prevents carbon migration into the substrate at elevated temperatures. Thus, the SiN_x_ underlayer effectively functions both as an adhesion layer and a diffusion barrier.

## Conclusions

The thermal stability and diffusion characteristics of ultrathin *a*-C films grown on crystalline and nitrogenated silicon substrates by the FCVA method were investigated by TEM and EELS. The nitrogenation of the silicon substrate surface by reactive RF sputtering using N^+^ plasma resulted in the formation of a 5-nm-thick SiN_x_ underlayer that prevented the migration of carbon atoms into the silicon substrate, while preserving the thermal stability of the *a*-C film. Cross-sectional TEM images revealed the growth of sub-5-nm-thick *a*-C films demonstrating desirable morphology and structure attributes, whereas the EELS spectra ascertained the respective *sp*^3^ fractions in the layered structure of the *a*-C films. The marginal decrease of the *sp*^3^ content of the bulk layer even after prolonged RTA confirmed the thermal stability of the *a*-C films in the *a*-C/Si and *a*-C/SiN_x_/Si stacks. While the effect of the RTA treatment on the thickness of the intermixing layer in the *a*-C/Si stack was secondary, an increase of the intermixing layer thickness in the *a*-C/SiN_x_/Si stack was found with the increase of the RTA time. This discrepancy is possibly due to structural differences between the intermixing layers of the two stacks. The subplantation process, which is intrinsic to film growth by the FCVA method, and the nitrogenation process used to form the SiN_x_ underlayer, which created pathways for carbon atom migration in the underlayer but not in the substrate, are the main physical processes responsible for the observed diffusion characteristics of the two stacks. The results of this study elucidate the important role of an ultrathin underlayer in heated layered media and provide an effective experimental framework for accurately assessing the thermal stability and elemental diffusion in ultrathin layered microstructures exposed to elevated temperatures.

## Methods

### Film deposition

A Si(100) wafer was cut in 5 $\times$ 5 mm^2^ substrates, which were then cleaned in acetone for 10–15 min. After drying the substrates in air, they were placed inside the chamber of an RF sputtering system (Perkin-Elmer, Randex 2400 model) and the chamber was outgassed at a vacuum pressure of ~10^–6^ Torr to remove any residual gases adsorbed onto the chamber walls. Subsequently, Ar gas was introduced into the chamber at a flow rate of 20 sccm, while the pressure was maintained at 3 mTorr, and the Si substrates were sputter-cleaned by Ar^+^ ion bombardment under conditions of 250 W RF forward power, 3 mTorr chamber pressure, and zero substrate bias voltage. Then, the chamber was pumped down again to remove any traces of Ar gas. After filling the chamber with N_2_ gas, ionization was instigated and the substrates were bombarded with N^+^ ions under conditions of 750 W RF forward power, 3 mTorr chamber pressure, 20 sccm N_2_ gas flow rate, and zero substrate bias for a duration of 3 min. Because of their small size, the energetic N^+^ ions were implanted into the near-surface region of the Si substrate forming a SiN_x_ layer.

Ultrathin *a*-C films were deposited on Si(100) substrates (with and without a SiN_x_ layer) using a custom-made FCVA system described in detail elsewhere^8, 9^. After pumping down the system to 10^–7^ Torr to remove any residual gases adsorbed onto the chamber walls, plasma arcing was initiated by striking a high purity (99.99%) graphite cathode with a mechanical striker. Plasma arcing was stabilized by a special cusp-configuration magnetic field generated in the vicinity of the cathode^9^. An electromagnetic coil with an out-of-plane S configuration was used to collect any macroparticles, expelled from the cathode during arcing, on the walls of the S-shaped filtering duct before they could reach the substrate. The C^+^ ions traveling through the applied magnetic field were imparted a certain radius of curvature (controlled by the ion mass and ion charge) by the Lorentz force acting perpendicular to both the C^+^ ion velocity and the direction of the magnetic field. During the deposition process, the current in the auxiliary, upstream, and downstream coils was set at 32 A, and the arc current was fixed at 80 A. Only C^+^ ions could reach the substrate surface under the former process conditions. The deposition of *a*-C films on bare and SiN_x_-covered Si substrates was performed under optimal FCVA process conditions of –80 V substrate bias voltage, 10° ion incidence angle (measured from the normal to the substrate surface), and 65% duty cycle of substrate pulse biasing^18–20^. To obtain ultrathin *a*-C films, the duration of each deposition was fixed at 6 s. During the deposition, the substrate was maintained at room temperature by water cooling and was continuously rotated at an angular speed of 60 rpm to enhance the film uniformity.

### Rapid thermal annealing

**Figure 7:**
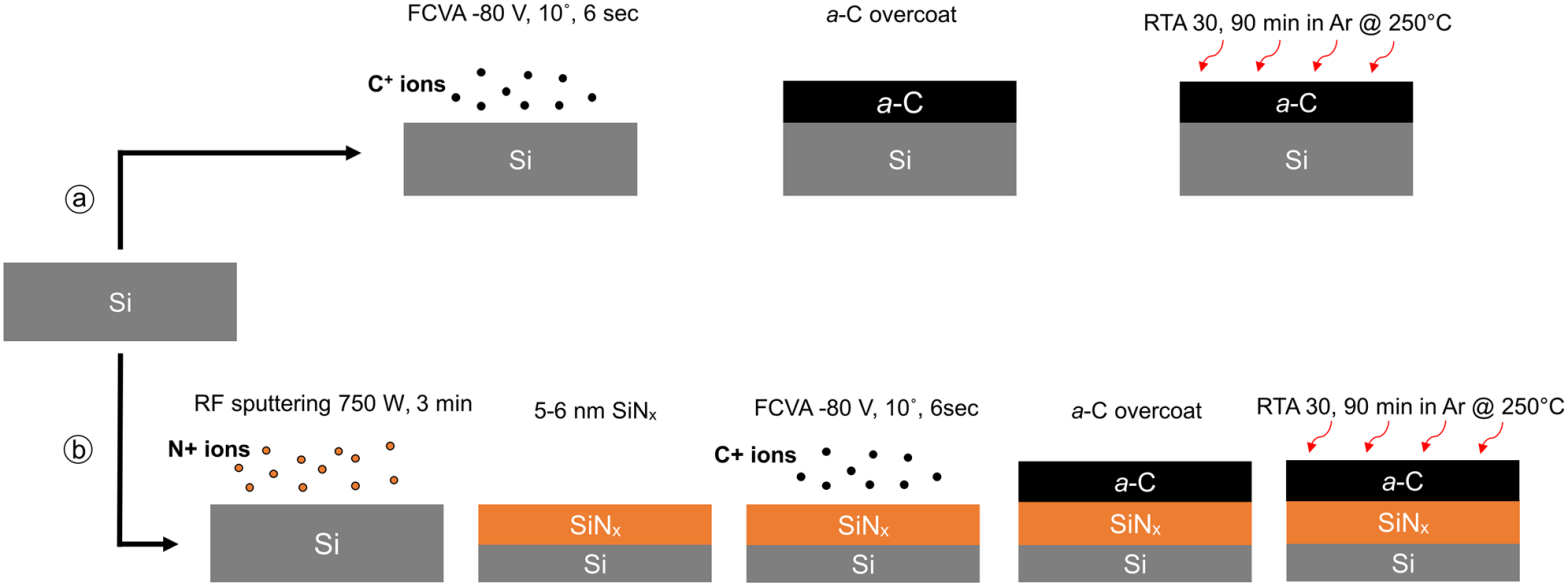
Schematic illustration of film synthesis by FCVA and RF sputtering deposition methods and heat treatment by RTA: (**a**) *a*-C film deposition on a Si substrate by FCVA followed by RTA and (**b**) SiN_x_ film formation on a Si substrate by reactive RF sputtering using N^+^ plasma followed by *a*-C film deposition by FCVA and subsequent RTA treatment under the same conditions as in (**a**).

Samples of the *a*-C/Si and *a*-C/SiN_x_/Si stacks were placed in the chamber of a heating system (AccuThermo, AW610 RTP) for RTA treatment. The process comprised heating from room temperature to 250 °C in 60 s, maintaining the temperature at 250 °C for either 30 or 90 min, and lastly, allowing the samples to cool down to the ambient temperature. To prevent oxidation during the heating and cooling cycles, a constant flow of Ar gas was maintained from the instant the samples were loaded onto the heating system until they were removed. The RF sputtering, FCVA, and RTA process steps used to fabricate and thermally treat the *a*-C/Si and *a*-C/SiN_x_/Si stacks are depicted schematically in Fig. 7.

### Microanalysis techniques

The fabricated samples were placed under an optical microscope and carefully wiped off with cotton tips to remove any adsorbents. The cleaned samples were then coated with a thin Cr capping layer to facilitate the distinction of the *a*-C film from the epoxy that was used to glue the samples and produce Si/*a*-C/Cr/epoxy/Cr/*a*-C/Si and Si/SiN_x_/*a*-C/Cr/epoxy/Cr/*a*-C/SiN_x_/Si stacks for the TEM, STEM, and EELS studies. The deposition of the Cr capping layer was performed with a thin-film ion beam coater (Gatan, Model 681 High Resolution Ion Beam Coater) using the following procedure. The samples were placed on a substrate holder covered with fresh filter paper and the vacuum chamber was pumped down to  ~3.75 $\times$ 10^–5^ Torr. The Cr target was then sputter cleaned for  ~1 min by Ar^+^ ion bombardment performed under conditions of 9 kV accelerating voltage and 425 µA ion gun current, followed by Cr deposition under the same conditions of accelerating voltage and ion gun current. Setting the deposition time to 10 min resulted in the growth of an approximately 25–30-nm-thick Cr layer. After the deposition of the Cr capping layer, the shutter was closed, the chamber was vented, and the sample holder with the mounted samples was removed.

The TEM/EELS samples were prepared by gluing the *a*-C surfaces of two samples face-to-face with M-bond 610 epoxy, allowing them to cure at  ~180 °C for 2 h, and finally, sectioning them in 1-mm-thick slices with a diamond blade. Sample curing did not affect the *a*-C film structure because the temperature was below the critical temperature (~200–250 °C) for activating *sp*^3^ → *sp*^2^ rehybridization^37^. The sliced pieces were then glued to a stub using crystal bond and thinned down in a polishing system (Allied High Tech, MultiPrep) using consecutively smaller diamond grits of size equal to 30, 15, 6, 3, 1, and 0.5 µm, and polishing one sample side until the slice thickness was reduced to 400 µm. At that juncture, the thinned sample was cleaned with micro-organic soap and heated on a hot plate to remove the sample from the stub. After this step, the sample was turned over and the opposite side was polished by the foregoing method until the thickness decreased to 100 µm. Then, the front edge of the sample was lowered to introduce the desired wedge angle by rotating the vertical adjustment knob of the polishing system. This process resulted in a flat slice with a 1° tip angle. The former wedge polishing technique produced a sample with a trapezoidal shape. Further polishing using this technique led to the formation of a wedge-shaped sample with one face completely flat and the other face sloped at 1° relative to the flat face. When the thinned slice attached to a transparent stub was observed under a microscope while light was transmitted through it, the thinned section appeared red in color. This wedge-like polishing technique was motivated by similar metallographic preparations reported elsewhere^38^. Finally, Ar^+^ ion milling (Gatan, PIPS 2) at a high energy of 6, 5, and 4 keV and ion incidence angle of 5°, 4°, and 3°, respectively, was used to further thin down the edge that contained the wedge, and the ion milling process was completed using ion energies of 2, 1, and 0.5 eV. The observation of fringe lines along the edge ensured that the sample was sufficiently thin for TEM, STEM, and EELS microanalysis.

High-quality TEM images and EELS spectra were obtained with a 200-kV FEI monochromatic (Tecnai, F20 UT) microscope (without a monochromator) equipped with a standard field emission gun. A 150-µm-diameter condenser aperture (C2) with a 9.3-mrad semi-angle and a 16.3-mrad collection semi-angle were used to acquire the EELS spectra. Using the full width at half maximum of the zero-loss peak, the energy resolution achieved in the EELS measurements was found to be in the range of 0.5–0.6 eV, which is adequate for distinguishing the *sp*^2^ and *sp*^3^ hybridization states because their bandgaps differ by 0.8–0.9 eV. A line scan starting from the Si substrate at the bottom of the stack, passing through the SiN_x_ underlayer and the *a*-C film, and terminating at the Cr capping layer at the top of the stack was used to collect sequential EELS spectra with a step size of  ~0.2 nm. A beam current of 80 pA and an acquisition time of 0.5 s were used in all of the line scan spectra. Two sets of EELS spectra were collected, i.e., a full survey spectrum of all the elements existing in the cross-sectional sample and a spectrum specific to carbon bonding.

The EELS technique is appropriate for analyzing the elemental composition and obtaining a better insight into the layered structure of the sample stacks. In this technique, incident electrons inelastically scatter inside the material, causing the electrons to lose energy and change travel paths^39^. By gauging this energy loss with an electron spectrometer, the ionizations of inner shells are used to determine the elemental components of the material. The ionization edge of carbon is at 285 eV and can be fitted with ${\pi }^{*}$ and ${\sigma }^{*}$ peaks in the range of 285–305 eV. Using the EELS spectra, the *sp*^2^ and *sp*^3^ hybridization fractions were determined by a method introduced in a previous study^13^ that uses the ${{\pi }^{*}/\sigma }^{*}$ ratio of the sample material, which is calibrated by the ${{\pi }^{*}/\sigma }^{*}$ ratio of a standard material. The standard specimen used to obtain reference spectra was graphitized evaporated carbon consisting of 100% *sp*^2^.

## Data Availability

The datasets generated and/or analyzed during the current study are available from the corresponding author on reasonable request.
